# The Impact of Microbiota on the Gut–Brain Axis: Examining the Complex Interplay and Implications

**DOI:** 10.3390/jcm12165231

**Published:** 2023-08-11

**Authors:** Tuba Shahid Chaudhry, Sidhartha Gautam Senapati, Srikanth Gadam, Hari Priya Sri Sai Mannam, Hima Varsha Voruganti, Zainab Abbasi, Tushar Abhinav, Apurva Bhavana Challa, Namratha Pallipamu, Niharika Bheemisetty, Shivaram P. Arunachalam

**Affiliations:** 1 Department of Neurology, Mayo Clinic, Rochester, MN 55905, USA; chaudhry.tuba@mayo.edu; 2 Department of Cardiology, Mayo Clinic, Rochester, MN 55905, USA; senapati.sidhartha36@gmail.com (S.G.S.); challa.apurva@mayo.edu (A.B.C.); 3 Department of Radiology, Mayo Clinic, Rochester, MN 55905, USA; drsrikanthgadam@gmail.com (S.G.); namrathapal123@gmail.com (N.P.); 4 GIH Artificial Intelligence Laboratory (GAIL), Division of Gastroenterology and Hepatology, Department of Medicine, Mayo Clinic, Rochester, MN 55905, USA; srisaimannam@gmail.com (H.P.S.S.M.); hima.voruganti@gmail.com (H.V.V.); zainab.abbasi@nghs.com (Z.A.); tusharabhinav.44@gmail.com (T.A.); dr.niharika16@gmail.com (N.B.); 5 Microwave Engineering and Imaging Laboratory (MEIL), Division of Gastroenterology and Hepatology, Department of Medicine, Mayo Clinic, Rochester, MN 55905, USA; 6 Division of Gastroenterology and Hepatology, Mayo Clinic, Rochester, MN 55905, USA; 7 Department of Medicine, Mayo Clinic, Rochester, MN 55905, USA

**Keywords:** enteric nervous system, central nervous system, gastrointestinal diseases, neurological disorders, CNS disorders, GI disorders, gut microbiota, serotonin, neurons, second brain, gastrointestinal mobility, cognitive development

## Abstract

The association and interaction between the central nervous system (CNS) and enteric nervous system (ENS) is well established. Essentially ENS is the second brain, as we call it. We tried to understand the structure and function, to throw light on the functional aspect of neurons, and address various disease manifestations. We summarized how various neurological disorders influence the gut via the enteric nervous system and/or bring anatomical or physiological changes in the enteric nervous system or the gut and vice versa. It is known that stress has an effect on Gastrointestinal (GI) motility and causes mucosal erosions. In our literature review, we found that stress can also affect sensory perception in the central nervous system. Interestingly, we found that mutations in the neurohormone, serotonin (5-HT), would result in dysfunctional organ development and further affect mood and behavior. We focused on the developmental aspects of neurons and cognition and their relation to nutritional absorption via the gastrointestinal tract, the development of neurodegenerative disorders in relation to the alteration in gut microbiota, and contrariwise associations between CNS disorders and ENS. This paper further summarizes the synergetic relation between gastrointestinal and neuropsychological manifestations and emphasizes the need to include behavioral therapies in management plans.

## 1. Introduction

**Figure 1:**
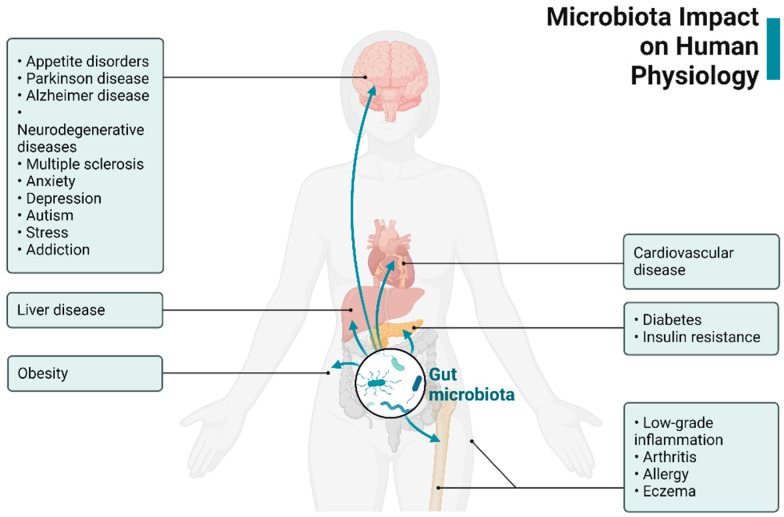
An illustration depicting the gut microbiome–brain axis. Created with BioRender.com.

Insights into the gut–brain system have revealed a multifaceted communication axis that ensures the adequate maintenance of gastrointestinal homeostasis [1]. The mechanisms underlying GBA communications involve neuro–immuno–endocrine mediators. New discoveries in the field have highlighted the significance of gut bacteria in affecting these interactions. Microbial products and microbially produced metabolites act as signaling molecules that have direct or indirect effects on the CNS and the ENS (Figure 1).

Recent research focuses on understanding the influence of the gut microbiome and its metabolites in neurodegenerative disorders like Alzheimer’s, Parkinson’s disease, MS, and even autism spectrum disorder [2]. In addition to metabolites, microbiota can produce a variety of neurotransmitters, including acetylcholine, histamine, norepinephrine, dopamine, gamma-aminobutyric acid, and serotonin [3]. Human intestinal microbes influence the gut–brain axis through various mechanisms and absorption of nutrients and, in turn, affect the central nervous system.

In this review, we provide an overview of the gut–brain axis, starting from embryological development to adult structure and function. We dive into gastro-electrophysiology and how the gut microbiome impacts its role in health, as well as in disease processes, including neurodegenerative diseases, mood, and motility disorders. In recent years, the development of rapid and credible sequencing technology has facilitated researchers and scientists to execute metagenomic investigations and studies, which have added a lot to our increased understanding of the host–microbe interface in health and disease. Sirtuins, a newly discovered class of molecules affecting gut homeostasis and metabolic diseases, are also discussed. Several other external factors affect gut homeostasis, including stress and feeding difficulties, the role of eating patterns, and their impact on cognition. Stress is considered a state of threatened homeostasis, usually resulting in various impacts on the GI tract, from impacting appetite to affecting GI motility, and playing a significant role in IBS; these concepts are also discussed.

Anatomically ENS has 2 main nervous complexes embedded in the wall: the myenteric plexus sandwiched between two layers of muscularized externa and the submucosal plexus, located in the submucosa. The enteric nervous system, often called the “second brain”, communicates with CNS via its sympathetic & parasympathetic fibers, working to fine tune the digestive functions [4].

The formation of ENS has been well studied in marine and avian embryo models [5]. Its formation starts with migration of a population of multipotent neural crest cells and differentiating within the walls of GI tract. The ENS attains functionality during the last trimester and continues to develop following birth [6]. Several growth factors affect this development and proliferation including but not limited to glial cell line-derived neurotrophic factor (GDNF), Sonic hedgehog, serotonin, neurturin and neurotrophin-3 [7]. Understanding of developmental processes also helps get insight into the process of aging, as several disease processes like Parkinson’s, vasculopathies and diabetes might lead to simultaneous aging in ENS. GI tract motility is initiated and regulated via biologic electrical activity termed as slow waves. The ionic concentration gradient across smooth muscle cells in coordination with interstitial cells of Cajal initiates a depolarization wave which is followed by contraction. Many motility GI disorders have been linked to disorders in these cells [8].

The gut-brain axis, which is defined as the two-way connection between the gastrointestinal tract and brain, which controls the central nervous system as well as intestinal homeostasis [9] This Axis connects the brain’s emotional and cognitive regions with the functioning of intestines at the periphery. The central nervous system, which comprises the brain and spinal cord, enteric nervous system, the autonomic nervous system, and the hypothalamus pituitary adrenal axis, are all parts of this two-way communication network.

New discoveries in the field have highlighted the significance of the gut bacteria in affecting these interactions. These bacteria have direct interactions with the CNS via neuroendocrine and metabolic pathways, in addition to local interactions with intestinal cells and the ENS. Microbiota interact with the gut-brain axis through a variety of methods, but the main one likely involves altering the intestinal barrier. Others being sensory afferents modulation, producing the local neurotransmitters, immune activation in mucosa, and by release of peptides. The brain also influences the gut microbiome via its efferents, by a mechanism that is dependent on the presence of neurotransmitters on the bacteria. It regulates the gut motility, secretions, mucus layer, permeability and immune function [10]. In this review, we provide an overview of the gut–brain axis, describing the CNS and ENS linkage both in healthy as well as in diseased conditions.

## 2. Materials and Methods

Electronic databases, including MEDLINE, EMBASE, PubMed, PsycINFO, CINAHL, and UpToDate, were searched for studies published up to and including December 2022. Reference lists of eligible studies were screened for further papers meeting the inclusion criteria. Databases were searched using a combination of the keywords, such as Enteric Nervous System, Central Nervous System, Gastrointestinal Diseases, Neurological disorders, CNS disorders, GI disorders, CNS, and ENS. Terms were based on MeSH indexing, as well as free text terms.

### 2.1. Microbiota

#### 2.1.1. Microbiota and Its Influence on ENS and CNS

The microbiota comprises a wide array of bacteria, viruses, fungi, and supplementary microorganisms co-existing within a single natural environment, such as the human digestive territory. The microbiome involves the whole locale of the body, which includes its microorganisms, genomes, and the adjoining ecological situations. The microbiota of the gut is a huge and complicated group of microorganisms that greatly impacts human health. Earlier, it was referred to as the microflora of the gut [11].

The total number of studies is constantly escalating, which shows that the microbiota of the whole digestive tract can be of a key role in the growth and maintenance of the CNS and the ENS. Before their own microbiota is obtained, eutherian fetuses are exposed to products and metabolites from the maternal microbiota. Colonization by microorganisms occurs at birth in infants. In the early stages of life, the microbial arrangement is much inspired by the way of delivery, the feeding method, the use of antibiotics, and the maternal microbial arrangement. Microbial products and microbially produced metabolites act as signaling molecules that have direct or indirect effects on the CNS and the ENS [12].

In recent years, the development of rapid and credible sequencing technology has facilitated researchers and scientists to execute metagenomic investigations and studies, which have impacted a lot to our increased understanding of the host-microbe interface in health and disease. Now the microbiota is established as an environmental component that affects the physiology of the host by playing vital roles in, for example, host immunity, metabolism, and behavior [13].

While we traditionally study by exploring the brain, many neurological conditions are impacted by input circuits from the periphery. In fact, promising data indicates an interaction between the gut and the brain in anxiety, depression, cognition, and autism spectrum disorder (ASD) [14].

The gut microbiota clearly seems to play a very vital position in the pathogenesis of Multiple Sclerosis (MS). It seems to be participating in regulating the immune system of the host, correcting the reliability and role of the BBB, prompting autoimmune demyelination, and is responsible for interacting precisely with different types of cells present in the CNS [15].

While mentioning neurological diseases, autoimmune-mediated encephalitis is a severe and system-specific destructive disease affecting approximately five to eight individuals per 100,000 persons. The gut microbiota we are describing, which is also referred to as the second brain, may influence brain activity because of the gut–microbiota–brain axis under both physiological and pathological circumstances. Dysbiosis (also called dysbacteriosis) in the gastrointestinal microbiota impacts proinflammatory T-cell variation and can stimulate autoimmunity, which is organ limited. The microbiota is also progressively linked to changes in features relevant to neurotransmission, including neurotransmitter signaling, synaptic expression of the protein, long-term potentiation, and myelination, in addition to a variety of complex host behaviors, including stress-induced, social, and cognitive behaviors [15].

#### 2.1.2. Microbiota and CNS

Significant research has shown that the microbiota can stimulate the gut tissue of the host and communicate with the brain in ways that affect the host’s behavior and the pathogenesis of neurological diseases. Over the past ten years, research has discovered a strong link between dysbiosis and a number of host diseases, including central nervous system disorders. 

Dysbiosis leads to the deregulation of the gut–brain axis pathways, which are connected to altered blood–brain barrier (BBB) permeability and inflammation of the nervous system. A variety of immunological mechanisms, including the inflammasome pathway, control both homeostasis and neuroinflammation. When a cell is activated by bacteria, danger signals, or stress, the inflammasome complex assembles, which causes the release of proinflammatory cytokines (interleukin-1 and interleukin-18), as well as programmed cell death (pyroptosis) [16].

Further investigation has revealed that the interaction between the microbiota and NF-B signaling is also responsible for CNS inflammation. The microbiome has an effect on the characteristics and operation of microglia, according to recent studies. Microglia have been shown to protect the brain against a range of illness states by triggering immunological responses that may rely on the interaction between GPR43 and inflammasome signaling. Like microglia, astrocytes play a variety of critical roles in the maintenance of CNS integrity. The generation of brain cytotoxic or immunological inflammatory chemicals, which cause CNS dysfunction and neurological diseases, is now known to be significantly mediated by excessive activation of astrocytes through IFN-1 signaling. A number of internal or external variables, including compounds produced by the gut flora that act on aryl hydrocarbon receptors (AHR) in animal models, might influence astrocyte activation.

Previous research suggests that gut microbes play an important role in regulating neurogenesis in the central nervous system, and that this intricate interaction mostly takes place in the hippocampus. Dysbiosis impairs memory in animal models by elevating NF-B activation and TNF expression. Conversely, restoring the microbiota’s composition reduces neuroinflammation in the hippocampus and improves pertinent symptoms. Studies indicate that some essential microbiota elements maintain the blood–brain barrier’s integrity, which in turn mediates the transfer of additional microbial signals from the gastrointestinal tract to the brain.

Following the discovery of a metabolic microbiota-derived molecule in the patient’s cerebrospinal fluid, recent research suggests that the gut microbiota is a key player in the etiology of Alzheimer’s disease (AD). Furthermore, it has been demonstrated that activated microglia cause an increase in Aβ deposition and an inhibition of Aβ clearance, both of which contribute to AD pathogenesis. Increased Aβ levels trigger the production of various proinflammatory molecules through microglia, causing neuroinflammation. 

An increase in local inflammation and ineffective clearance of alpha-synuclein deposits, which are caused by a persistent connection between microbial metabolism and TLRs, combine to further aggravate the neurodegeneration associated with Parkinson’s disease (PD).

The involvement of gut dysbiosis in the severity of the disease has been thoroughly supported by research. Changes in the abundance of specific Enterobacteriaceae species are linked to changes in the severity of symptoms, including aberrant gait and postural instability. Additionally, a decrease in Lachnospiraceae causes PD patients’ motor and nonmotor symptoms to deteriorate severely [17].

#### 2.1.3. Microbiota and ENS

The microbiota of the gut controls the metabolism of amino acids, lipids, and carbohydrates; all the substances are crucial for maintaining human health and preventing metabolic disorders [18]. The microbiota of the gut influences the metabolic nature and health by secreting short-chain fatty acids (SCFAs) by the fermentation of carbohydrates. The important SCFAs are formate, propionate, butyrate, and acetate, which are involved in maintaining the integrity of intestinal epithelium and its permeability [19]. According to reports, intestine metabolic regulation involves the enteric nervous system (ENS), and ENS regulation is significantly affected by intestinal neurotransmitters and enteric neurons [20]. The CNS and ENS interact via the vagal nerve route, which has a strong influence on modulating gastrointestinal functions and feeding behavior [21]. Therefore, through the gut–brain axis, the vagal nerve system also influences metabolic regulation in the intestine. Gut peptides, such as leptin, cholecystokinin (CCK), peptide tyrosine tyrosine (PYY), ghrelin, glucagon-like peptide-1 (GLP-1), leptin, 5-hydroxytryptamine (5-HT), and others that are produced by enteroendocrine cells, have receptors that are located on vagal afferent neurons (EECs) [22]. These kinds of peptides are detected by vagal afferent neurons, which transfer their respective gut information to the central nervous system and cause diverse reactions. At the same time, gut bacteria can influence the levels of gut peptides, such as leptin, CCK, PYY, ghrelin, GLP-1, and 5-HT, to regulate the vagal afferent output. Then, the microbiota can regulate intestinal metabolic metabolism [23,24,25]. Therefore, it has been demonstrated that the microbiota of the gut affects the release of 5-HT from EECs, which in turn activates 5-HT3 and 5-HT2 receptors present in vagal afferent neuronal cells [26]. In addition to influencing the vagal afferent pathway, lipopolysaccharides (LPS) secreted by Gram-negative bacteria in the colon can also lead to inflammation and obesity. Specifically, LPS can be recognized by the TLR4 receptor located on vagal afferent neurons, which then sends the signal to the brain [27,28].

Apart from metabolites, microbiota can produce a variety of neurotransmitters, including acetylcholine, histamine, norepinephrine, dopamine, gamma-aminobutyric acid, and serotonin [3]. Alteration in the microbiota of the gut, inflammation, and host stress can all have an impact on the anatomy and functioning of the intestinal mucosal barrier. It has been revealed that altered microbiota and a disrupted intestinal barrier have a significant influence on the emergence of many diseases, which include a variety of inflammatory disorders, mental disorders, and metabolic abnormalities [29]. The BBB functions as a gate for information and the exchange of nutrients between the circulation and the parenchyma of the brain. Also, several substances secreted by gut bacteria can cross the BBB [30].

### 2.2. CNS Disorder and Their Gi Consequences

Even though the interaction between the enteric nervous system and the central nervous system is well-established scientifically, the gastrointestinal symptoms in central nervous disorders are not emphasized enough, resulting in delays in the diagnosis and management of the underlying disorder. The nervous system disorders seem to mostly result in gastrointestinal motility disorders rather than disorders of secretory function [30]. The entire neural axis, from cerebral hemispheres to peripheral autonomic nerves, can affect the enteric nervous system. Even though there are a significant number of neurological diseases causing GI consequences, the most common CNS disorders are stroke, parkinsonism, multiple sclerosis, and diabetic neuropathy.

The pathophysiology of involvement of the enteric nervous system in various neurological disorders is mainly determined by the underlying neurological disorder. However, most neurological disorders have similar motility-related gastrointestinal symptoms. The common symptoms in most of the CNS-related disorders, dysphagia, constipation, diarrhea, intestinal pseudo-obstruction, dysphagia, gastroparesis, nausea, vomiting, sialorrhea, and fecal incontinence are elaborated regarding their etiology, pathogenesis, and management in this section.

Dysphagia is most commonly seen with stroke, with significant other etiologies including Parkinson’s disease, Alzheimer’s disease, motor neuron disease, multiple sclerosis, head trauma, and polymyositis [31,32,33]. Any disruption of the semi-autonomous swallowing pathway from corticobulbar fibers to efferent nerves innervating the muscles can result in neurogenic dysphagia. A multidisciplinary approach is needed for rehabilitation with swallowing therapy and gastrostomy, addressing potential complications like malnutrition and aspiration pneumonia, insufficient drug effects, and treatment of the underlying neurologic condition [33,34].

The most commonly neglected symptom in neurological disorders is constipation. Constipation in neurological disorders negatively impacts one’s quality of life, reduces their capacity to engage in social activities, causes poor neurological function, or even causes death. It is seen in 1 in 3 patients with acute stroke, 1 in 2 patients with Parkinson’s disease [35], 1 in 2 patients with multiple sclerosis, and in around 60 percent of diabetes mellitus patients. Constipation also occurs commonly in patients with multiple system atrophy, spinal cord disorders like cauda equina lesions, and spinal cord injury [36]. Underlying pathologies causing constipation include impaired peristaltic wave synchronization, damage to the pontine defecatory center, and immobility and hypovolemia in acute conditions. Other causes include poor pelvic floor relaxation, degeneration of dopaminergic neurons of the myenteric and submucosal plexus. Constipation can be corrected by addressing underlying neurological disorders, lifestyle changes, tailoring laxatives, and increasing fiber intake [35].

Another symptom of neurological disorders, intestinal pseudo-obstruction, is characterized by the dilation of the bowel in the absence of an anatomical obstruction. It can be present as acute or chronic. Acute pseudo-obstruction is usually the neurological consequence seen in critically ill and postoperative patients [37]. Chronic pseudo-obstruction is a rare form of intestinal pseudo-obstruction in adults, usually seen secondary to neurologic and other disorders [38]. Chronic intestinal pseudo-obstruction presents as constipation, recurrent episodes of abdominal pain, abdominal distention with or without nausea, vomiting, and diarrhea. It is often misdiagnosed due to sub-occlusive episodes. Some of the neurological causes of pseudo-obstruction are pandysautonomia, pure autonomic failure, stroke, encephalitis, calcification of basal ganglia, myasthenia gravis, and autoimmune neuropathy [39,40]. Underlying processes include reduced ganglion cells, dysregulated stretch receptors, and suppression of parasympathetic action in the colonic smooth muscle [41,42]. It is treated as a mechanical obstruction in acute cases with prokinetics, octreotide, and nutritional support. The management of complications like bowel ischemia, intestinal perforation, and sepsis is important to prevent adverse events [41,43].

Gastroparesis should be kept in mind when patients with neurological disorders present with typical or atypical symptoms, such as abdominal pain, nausea, vomiting, and postprandial fullness [44,45]. Gastroparesis is often seen in patients with Parkinson’s disease and diabetes mellitus. It is less commonly seen in patients who have undergone vagotomy, and in patients with vagus nerve damage during fundoplication. Gastroparesis is often reported in patients with myasthenia gravis, Guillain–Barre syndrome, and amyotrophic lateral sclerosis [44]. It is reported that there is a reduction in nitrergic inhibitory neurons, neuronal dysplasia, and a significant decline in Cajal interstitial cells in patients with gastroparesis. Dietary changes, prokinetics, antiemetics, and neuromodulators are found to be most helpful in patients with gastroparesis [44,46]

Nausea and vomiting are frequently associated with neurological disorders [47]. Increased biosynthesis of emetic neurotransmitters from the gastrointestinal tract and brainstem is thought to play an important role. It is usually managed by addressing underlying neurological disorders and providing supportive therapy with antiemetics.

#### 2.2.1. Neurodegenerative Disorders and Involvement of ENS

Inflammatory and neurodegenerative disorders can also have an effect on ENS, significantly affecting the quality of living for people. The gastrointestinal tract is greatly affected by Parkinson’s disease, with oropharyngeal dysphagia and esophageal dysphagia, anorectal dysfunction, gastroparesis, small intestine and colonic dysmotility, hypersalivation, in addition to the neurologic manifestations caused by the loss of dopaminergic neuronal cells of the central nervous system, with severe deterioration of motor activity [48,49,50]. Numerous investigations over the last 40 years have revealed a neurodegenerative pathophysiology in the gastrointestinal tract that is comparable to that in the central nervous system. To be specific, enteric dopaminergic neuronal loss and the presence of Lewy bodies and synuclein deposits in enteric cells—markers for neuronal degeneration in Parkinson’s disease—were discovered [51]. In bioptic and postmortem studies on patients with constipation-related Parkinson’s disease, fewer dopaminergic neurons were found in the colon. In certain cases, less amount of dopamine was detected using HPLC, i.e., High-Performance Liquid Chromatography in muscularis externa [52]. Phosphorylated-synuclein immune-positive neuritis was discovered in patients’ submucosal ganglia during routine colonic biopsies [53]. More recently, conventional staining with H&E, i.e., hematoxylin/eosin, was carried out on the jejunum and colon of certain patients who passed away from Parkinson’s disease, revealing the existence of atrophic or pyknotic neuronal cells in both submucosal and myenteric plexuses. Additionally, deposits of synuclein were seen with degenerative changes and in some intact neurons, indicating that the buildup of synuclein occurs before neurodegeneration [54]. Dementia with Lewy bodies or Parkinson’s disease patients have synuclein aggregates found in their brains or GI tracts. Protein misfolding and cyclic amplification of the real-time quaking-induced conversion assays were used to find this [55]. Appendectomy appears to be related to a lower chance of developing Parkinson’s, and it has been demonstrated that the appendix contains a high number of collapsed aggregates of synuclein that promote protein spreading [56]. In the myenteric plexus of adult rats, native synuclein is thought to be present in high concentrations. Nevertheless, this protein is a potential diagnostic for neuropathies in old animals due to its tendency to misfold and aggregate, which results in synuclein aggregates. In this regard, early middle-aged and aged rats’ GI tracts were discovered to include synuclein-positive dystrophic axons, which are co-localized with either NO synthase, calbindin, or Tyrosine Hydroxylase-reactive swelling neurites and calretinin. These results point to a temporal link between the initiation of degeneration and the buildup of misfolded proteins like synuclein [57]. It should come as no surprise that a substantial body of research points to enteric glial cells as having a role in the pathophysiology of inflammatory and neurodegenerative GI illnesses, such as Parkinson’s disease. Parkinson’s patients have increased levels of Sox-10 glial markers and GFAP in their GI tracts, which are correlated with inflammatory cytokines. This, most of the time, is seen at the beginning of the condition, but it gradually gets less severe. Since GFAP phosphorylation is thought to be crucial in CNS illnesses, reports of phosphorylated GFAP at amino acid serine 13 in colon biopsies of patients have been made. As it was not observed in patients with progressive supranuclear palsy or multiple system atrophy, this reactive gliosis appears unique to Parkinson’s disease [58,59].

Alterations to the microbiota of the gut and epithelial barrier in the intestine can cause neuroinflammation in enteric glial cells, which can lead to the evolution of Parkinson’s disease and the concentration and aggregation of alpha-synuclein. Then, alpha-synuclein might spread to the brain via a process involving glial cells to glial cell interactions in a prion-like manner [60]. Furthermore, it has recently been discovered that Triggering Receptors Expressed on Myeloid Cells (TREMs), particularly TREM-1, are crucial in the inflammatory changes affecting the gastrointestinal tract and the microbiota–gut–brain axis, which may be significant in the etiology of neurodegenerative disorders [61]. The pathophysiology of alpha-synucleinopathies is also thought to include malfunctioning of Toll-like receptor signaling [62]. Alterations in the synthesis of these receptors and alterations in cell-clearing processes like proteosome and autophagy may be linked to inflammation brought on by intestinal barrier dysfunction and microbial dysbiosis [61]. In addition, fecal microbiota transplantation, probiotics, diet, and nutritional supplements have been proven to delay the clinical advancement of Parkinson’s disease [63]. This makes the Enteric Nervous System a possible therapeutic target for many gastrointestinal illnesses [64].

#### 2.2.2. Serotonin

Serotonin or 5-hydroxytryptamine(5HT) is one of the most versatile monoamine neurotransmitters with functions ranging from modulating memory, mood, reward, pain, sleep, and learning to various physiological processes such as vomiting and vasoconstriction [65,66]. Most of the serotonin is synthesized in the intestine and is synthesized by enterochromaffin cells (ECs) [65]. Multiple studies have suggested the role of gut microbes in central serotonin homeostasis [3,67].

There are around 15 serotonin receptors which are virtually expressed outside as well as inside the brain, with most of the serotonin being found outside the central nervous system (CNS) [68]. As serotonins have been found to be playing an important role in the development of the enteric nervous system (ENS) and CNS, their low levels have been implicated in defects in ENS development and GI motility, and increasing levels of 5HT by feeding chow was seen to reduce depressive-like behaviors in models and to improve ENS, gut motility, and enteric epithelial growth [3,65,67,68,69]. A link was found between constipation and mood dysfunction through neuron production of 5HT [69]. Deficient levels of serotonin are also known to be associated with schizophrenia and autism [70].

#### 2.2.3. Autism and Serotonin

The association of autism with abnormal serotonergic function was first put forth when Schain and Freedman discovered elevated serotonin levels in the blood of autistic children [71]. About 33% of autistic children have hyperserotonemia [72]. This finding has been replicated and expanded with the discovery of hyperserotonemia in the first-degree relatives of these autistic children [71]. New evidence from postmortem samples and neuroimaging studies both point to alterations in the brain’s serotonin pathway in ASD [73]. Two distinct types of serotonergic abnormalities were discovered in studies. The first is a variation in how serotonin synthesis throughout the brain changes with age. In other words, a child with autism has a much lower capability for serotonin synthesis at a given young age of less than 5 years than a child without autism [71]. It is believed that altered serotonin levels during early childhood led to aberrant brain circuitry and symptoms of autism [72]. In a tiny sample of children with autism, the second type is found, which is a localized abnormality in the brain’s serotonin synthesis uptake in the thalamus, cerebellum, and frontal cortex [71].

Numerous attempts have been made to find genetic variations that increase autism susceptibility due to the high heritability of the disorder. Anderson et al. study findings imply that minor differences in the serotonin pathway genes, particularly HTR3A on chromosome 11, may have an influence on the chance of developing autism [74]. Another team has identified a susceptibility mutation in the tryptophan 2,3-dioxygenase gene’s promoter variant [71]. According to studies, selective serotonin reuptake inhibitor therapy reduces some of the symptoms of autism by interacting with serotonin transporters. The expression of the transporter (SLC6A4) is also known to be modulated by variations in the gene encoding that protein, particularly at the HTTLPR locus (serotonin-transporter-linked promoter region) [75]. Results of the study conducted by Tordjman S. et al. show that HTT promoter alleles alone do not increase the risk of autism; rather, they affect how severe autistic behaviors are in the social and communicative domains [76].

#### 2.2.4. Nutrients and Cognition

The human brain requires complex interactions between genetic and environmental elements to function properly. Together with them, experience influences the structure and functioning of the brain, influencing neuroplasticity events like neurogenesis, synaptogenesis, and spinogenesis [77]. Research has shown that eating patterns cause alterations in the brain that have an impact on cognition [78,79,80]. A healthy diet prevents age-related cognitive impairment by altering a variety of biological processes, including those that control the fluidity of neuronal cell membranes, synaptic plasticity, neuroinflammation, oxidative stress, neuroprotection, and neurogenesis [77]. Many nutrients, through epigenetic mechanisms, modify the central nervous system and affect cognition [81]. Along with N-3 PUFAs, polyphenols, vitamins B, D, and E are essential for maintaining cognition in older people and minimizing cognitive decline [80,82,83,84,85]. The influence of nutrients on cognition is known to happen as early as the periconceptional period and during pregnancy [86]. Human intestinal microbes influence the gut–brain axis through various mechanisms and absorption of nutrients and, in turn, affect the central nervous system [77].

### 2.3. GI Diseases and Their Associations with CNS

#### 2.3.1. Inflammatory Bowel Disease and CNS Manifestations

In this section, we aim to briefly discuss the neurological manifestations of inflammatory bowel disease. Neurologic involvement in IBD can emerge before or after a diagnosis of inflammatory bowel disease. Additionally, neurological symptoms could become more severe during IBD flare-ups or develop separately from intestinal manifestations that do not resolve with the treatment for the underlying bowel disease [87]. In inflammatory bowel disease, the malabsorption of nutrients and vitamins, dysfunctional immune system, infections, side effects of medications (metronidazole, sulfasalazine, steroids, cyclosporine), iatrogenic complications from intestinal surgery, and pre-coagulation/thromboembolic state are the possible underlying mechanism for neurological manifestations [88]. The common neurological manifestations include Cerebrovascular disease, Cerebral infarction, transient brain ischemia, cerebral venous thrombosis, demyelinating disease, Melkersson–Rosenthal syndrome, multiple sclerosis, optic neuritis, Sensorineural hearing loss, headache, neuropathies, myositis, epilepsy, psychosis, major depression, chorea, vasculitis, restless leg syndrome [89].

#### 2.3.2. Irritable Bowel Syndrome and ENS

Intrinsic primary afferent neurons (IPANs) and extrinsic primary afferent (sensory) neurons’ mucosal processes are activated by sensory transducers in the epithelial cells, such as enterochromaffin (EC) cells. Serotonin (5-HT) is secreted by EC cells in feedback to mucosal stimuli. Calcitonin gene-related peptide and acetylcholine (Ach) are secreted by submucosal IPANs, which are triggered by “5-HT1P” receptors to cause peristaltic and secretory reflexes. 5-Hydroxytryptamine receptors, which are usually presynaptic and enhance neurotransmission in prokinetic pathways, increase the release of neurotransmitters. IBS-D with diarrhea as its major symptom is alleviated by 5-HT3 receptor-mediated signaling to the CNS; however, because 5-Hydroxytryptamine receptors also influence fast ENS neurotransmission and charge the myenteric IPANs, they may have constipating effects. In IBS-C and chronic constipation, 5-HT4 agonists are prokinetic and therefore reduce constipation. Instead of starting peristaltic and secretory reflexes, 5-HT4 agonists augment already active pathways. SERT, a transmembrane 5-HT transporter, shuts down serotonergic signaling in the ENS and mucosa. In IBS-C, ulcerative colitis, and IBS-D, the expression of tryptophan hydroxylase-1 and mucosal SERT is reduced. IBS-D pain and diarrhea may be caused by enhancement of 5-HT brought on by SERT reduction, whilst constipation may result from receptor desensitization. SERT-deficient transgenic mice have symptoms that are similar to these. Therefore, the pathophysiology of IBS may be influenced by the loss of mucosal SERT [90].

#### 2.3.3. Sirtuins-Interlinking ENS, CNS and Beyond

Sirtuins, also known as silent information regulators, are a newly discovered class of molecules that, according to recent studies, are being stated as one of the key regulators in multiple processes in the human body—ranging from mood, satiety, circadian rhythm, and gut homeostasis to inflammatory signaling, responses to aging, and metabolic diseases [91]. Sirtuins belong to the family of class III histone acetylases, having a conserved catalytic domain of 275 amino acids. They require NAD+ for their enzymatic activities, and it has been seen that an increase in levels of NAD+, caused by calorie restriction, can activate sirtuins [91,92]. As of now, 7 types of sirtuins have been discovered—namely SIRT1–7. All seven sirtuins can be found in almost all human tissues, with one type being more abundantly present in certain specific areas than another. SIRT1, SIRT6, and SIRT7 are found in the nucleus, whereas SIRT3, SIRT4, and SIRT5 are found in the mitochondria, with SIRT2 being found in the cytosol. SIRT1 and SIRT2 are the most widely expressed sirtuins subtypes in neurons of CNS, with SIRT1 expression being highest in the cerebellum, hippocampus, and hypothalamus and SIRT2 expression being highest in the spinal cord and the brain stem. SIRT3, SIRT4, and SIRT5 are expressed in CNS at a level lower than SIRT1 and SIRT2 but higher than SIRT6 and SIRT7. SIRT7 has been found to have 10 times higher expression in the small intestine than all other sirtuins. Expression of SIRT1 and SIRT3 have been detected by recent studies in neurons of ENS [91,93].

The whole family of sirtuins plays an important role in orchestrating numerous physiological processes involving the central nervous system (CNS) and enteric nervous system (ENS). Recent studies have shed light on the importance of sirtuins in regulating neurodegeneration associated with toxic traits of aging, neurodegenerative diseases, obesity, and diabetes. One fascinating thing to note is that SIRT1 and SIRT6 show decreasing and increasing trends, respectively, with aging. Overexpression of SIRT1 has been shown to have a protective effect on neurons against neurodegenerative diseases such as Alzheimer’s disease (AD), whereas the same does not hold true for SIRT2, whose overexpression increases the susceptibility of AD, and inhibition of SIRT2 has shown better outcomes in the preclinical Parkinson’s disease model. SIRT3 and SIRT5 have been shown to have maximum protective efforts against nigrostriatal pathway neurons. SIRT6 also showed an important role in maintaining genomic instability and keeping the brain healthy. Many agonists of sirtuins, such as resveratrol, and quercetin, have shown promising results in preventing cell death in striated neurons seen in amyotrophic lateral sclerosis (ALS), spinal muscular atrophy (SMA), and Huntington’s disease [91].

SIRT1 has also been shown to protect against the harmful effects of a high-fat diet, and its inhibition in the hypothalamus leads to an increase in neurons promoting satiety via the expression of proopiomelanocortin and agouti-related peptide. SIRT3 deletion was seen to be associated with impaired cognition in mouse models [91].

In the ENS, there has not been the same number of observations as compared to CNS, demonstrating a requirement for further study. SIRT1 has been found to be playing a pivotal role in regulating food intake and circadian rhythm, and it is also evidenced as a potential target in inflammatory diseases of the intestine. Surprisingly, the protective role of SIRT3, relative to its role in CNS, was not seen in ENS [91].

Sirtuins, especially SIRT1, also play a role in regulating the gut microbiota, which takes part in facilitating the complex conversation between the brain and gut. Studies have found evidence of recognizable alterations in gut microbiota composition in CNS disorders. Resveratrol, which activates SIRT1 through a complicated process, has been proposed to possess antibacterial properties and also increase the expression of intestinal tight junction proteins improving epithelial barrier integrity. Thus, maintaining the gut microbiota consisting of Firmicutes, Bacteroidetes, and Actinobacteria [92].

#### 2.3.4. Gastrointestinal Functional Disorder and MOOD Disorders

In recent times, there has been increasing emerging interest in understanding the factors that contribute to diseases that involve both central and enteric nervous systems. In recent times Serotonin has been determined as a major contributor to brain-gut conditions [94]. About 95% of serotonin is produced in the intestine, where it also has paracrine and endocrine functions [95]. 5-HT is essential for the growth and performance of both ENS and CNS. Mutations in 5-HT affect the development and function of these organs, like GI motility, mood, and behavior [96,97,98]. Polymorphism of enzymes involved in serotonin synthesis and homeostasis has been associated with disease states involving both systems (105–108). The most common GI problems reported in ASD are constipation, diarrhea, and Gastroesophageal reflux disease (GERD) [99,100]. In the biosynthesis of 5-HT in serotonergic neurons of both systems, TPH2 is the rate-limiting enzyme that is critical. The polymorphisms in TPH2 have been identified to occur in a variety of conditions in both systems. For example, depression [100,101,102] and anxiety [103,104] are co-morbid with Gastrointestinal (GI) disorders like constipation and irritable bowel syndrome (IBS) [104]. The converse is also reported where IBS and constipation can co-occur with psychiatric conditions like anxiety and depression [105]. One of the TPH2-related single nucleotide polymorphisms where an arginine is replaced with histidine was identified in a cohort of patients with unipolar depression (TPH2-R441H) [106]. Hypo-efficiency to TPH2 is reported in those who have TPH2-R441H polymorphism, and interestingly, the patients in this cohort experienced worsened depressive states with an SSRI [106]. Using the 5HT-mediated pathways should be considered as a goal for future therapies.

#### 2.3.5. Stress-Induced GI Disorders

Stress is considered a state of threatened homeostasis, usually resulting in a process of inappropriate response to physical or emotional threats [107]. Stress is known to impact the gastrointestinal tract by altering gut motility, and intestinal motility, increase in visceral perception, mucosal permeability, mucosal barrier function, and blood flow to the mucosa, decreasing regenerative capacity of the gastrointestinal mucosa, increasing GI inflammation, negatively affecting intestinal microbiota, down-regulate different immune system components and altered perception of sensory input in the central nervous system [108].

The effects of stress on GI can be divided into 4 categories: impact on appetite, motility dysfunction, digestive dysfunction, and GI inflammation [109]. Here, we aim to discuss the common GI disorders seen with stress, including functional GI disorders and inflammatory bowel disease [108,110,111]. The evaluation of mental health in these patients remains a key factor in the management of these conditions.

Functional gastrointestinal disorders encompass various gastrointestinal (GI) symptoms (e.g., abdominal pain, dysphagia, dyspepsia, bloating, and diarrhea, constipation) without demonstrable pathology on conventional testing. They include 33 different adult disorders affecting the different parts of the GI tract, with irritable bowel syndrome and functional dyspepsia being the most common [112]. Irritable bowel syndrome (IBS) patients experience chronic or recurring abdominal pain with altered bowel motility, and are diagnosed using ROME 4 criteria [113]. Chronic stress and IBS have bidirectional effects on each other, with symptoms deteriorated by maladaptive patient behaviors, stress, and underlying psychological disorders [114]. Patients with normal physical examination meeting the criteria for IBS should be advised of lifestyle and diet changes, addressing the underlying psychological condition, assuring the patient (in the absence of warning signs) to establish a good therapeutic relationship and medical management with antispasmodics, neuromodulators, motility agents and antidepressant [115]. In functional dyspepsia (FD), patients present with epigastric pain or discomfort, and postprandial fullness, often associated with food without structural disease in imaging or endoscopy [112]. Management mainly includes proton pump inhibitors and eradication of Helicobacter pylori infection, prokinetics, tricyclic antidepressants, and psychotherapy [116].

Inflammatory bowel disease (IBD) has diverse chronic and relapsing manifestations, including abdominal pain, weight loss, and bloody stool. Of the various genetic and environmental factors that are associated with inflammatory bowel disease, stress is noted to act as a promoting or relapsing factor in these patients [117]. Stress is likely to interrupt the normal hypothalamic-pituitary-adrenal axis, alter gut microbiota interaction, and mucosal mast cell activation, and increase the release of corticotrophin-releasing factor, resulting in exacerbation of symptoms of IBD. IBD has a bidirectional relationship with stress and therapeutic interventions, so interventions like cognitive behavioral therapy (CBT), medical hypnosis, meditation, and antidepressants are helpful in addition to aminosalicylate agents, oral glucocorticoids and immunomodulators [118,119].

## 3. Discussion

The Enteric nervous system (ENS) consists of a network of neurons and glial cells present along the length of the gastrointestinal tract. There exists a bidirectional communication between ENS and CNS through a complex network of neural pathways and mediators (neurotransmitters, hormones, neuropeptides, hormones, and immune cells), which has a significant implication on the regulation of GI function and overall health. Many studies have shown an association between infections and neurodegenerative diseases. However, the mechanisms by which the gut microbiome plays a neurophysiological role are not clearly understood. For instance, the association between H. pylori and Parkinson’s disease (PD) is linked to reduced absorption of levodopa [120]. Similarly, the prevalence of gastrointestinal dysfunction and small intestinal bacterial overgrowth (SIBO) in PD and AD [121]. With the advent of metagenomics, metabolomics, and other sequencing techniques, a slight deviation in gut microbial composition has been observed in PD and AD. Suppression of growth of anti-inflammatory bacteria such as Faecalibacterium, Roseburia Coprococcus, Prevotellaceae, and Blautia with a profusion of proinflammatory bacteria Enterobacteriaceae, Proteobacteria, and Enterococcaceae [122].

Gut infection, SIBO, and dysbiosis are associated with a disrupted gut barrier, overactivation of the inflammasome, and release of inflammatory cytokines leading to systemic inflammation (Table 1). The blood–brain barrier (BBB) disruption from systemic inflammation, initiating the neuroinflammatory response and altering homeostatic mechanisms. Moreover, other factors that may exacerbate the existing condition include poor diet, physiological changes from aging like increased inflammatory response, decreased neurotransmitters, and increased oxidative stress [122]. Apart from increasing gut permeability and release of interleukins, bidirectional communication exists between ENS and CNS via the vagus nerve, neurotransmitters (Serotonin), and metabolites (SCFA) [9,123]. IL-18 has been seen to be associated with the maintenance of homeostasis in the gut [9]. Gut inflammasome activation may result in the release of effector molecules that may affect the CNS mediated through the vagus nerve. Inflammasome activation is associated with various neuroinflammatory conditions, resulting in the release of proinflammatory cytokines like IL-18 and IL-1 β, and pyroptosis. Innate immune cells of the brain, like oligodendrocytes, astrocytes, cells, perivascular resident macrophages, and endothelial cells, also show inflammasome-mediated inflammatory activity [16].

Genetics, endocrine cells, gut microbiota, and low-grade inflammation interact with each other in the pathophysiology of IBS. Studies on experimental animals have shown an association between the alteration of gut microbiota and gastrointestinal changes like gastrointestinal dysmotility, visceral hypersensitivity, and permeability, which is similar to the findings in IBS [131]. Alteration of gut flora is not just associated with gastrointestinal symptoms but also psychiatric symptoms prevalent in IBS patients [131]. Hormones released from the gut play a significant role in the pathophysiology of IBS by affecting visceral hypersensitivity, gut dysmotility, and abnormal secretion [132]. The choice of food items depends on the intestinal bacterial profile.

The gut microbiota’s role in the pathophysiology of IBD is not very clear; however, IBDs response to antibiotic treatment raises suspicion about the intestinal microbiome’s contribution to the inflammatory response. Studies have shown changes in gut microbial composition with a reduced proportional abundance of *Faecalibacterium prausnitzii* in the stools of patients with IBD and the rise of Enterobacteriaceae [133]. Studies have shown the prevalence of psychological morbidity in IBD patients during longitudinal follow-up. Treatment targeted towards centrally acting mechanisms has shown functional improvement in patients with IBS and functional dyspepsia [134,135]. Moreover, it has also been seen pre of psychological disorders in inflammatory conditions [136,137].

**Figure 2:**
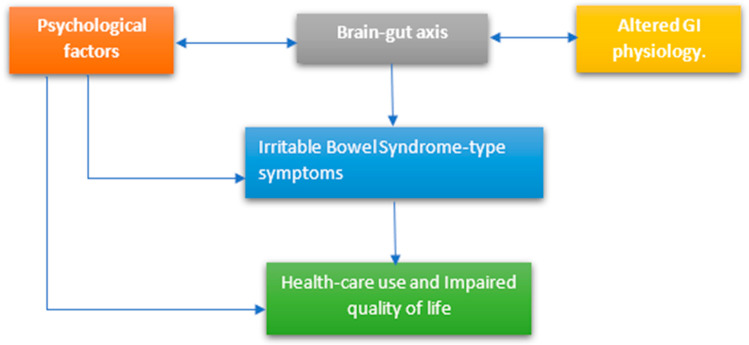
Biopsychosocial model for IBD [144].

In IBS, activation of the immune system by bacterial lipopolysaccharides, consequent brain-gut axis activation through afferent sensory nerve stimulation, may also be relevant in the development of IBS-type symptoms. This may also explain the prevalence of psychological disorders, anxiety, and depression, up to 35% in IBD patients [138]. Psychological disorders may induce stress response resulting in the activation of the hypothalamus-pituitary-adrenal axis, the release of adrenocorticotropic hormones, increasing intestinal permeability, and proinflammatory effects on the gastrointestinal tract mediated through macrophages and mast cells, and releasing C-reactive protein [139,140]. Treatment through vedolizumab (anti-TNFα therapy) has been seen to improve depression scores [141]. Antidepressants might prove to be beneficial through two different pathways: (1) acting on anti-inflammatory effects mediated by the vagus nerve; and (2) directly acting on nitric oxide pathways and factor-κB responsible for the generation of proinflammatory cytokines [142]. Gut-directed hypnotherapy and cognitive behavioral therapy (CBT) have been proven to be beneficial in patients with IBS (Figure 2) [143]. Neurodegeneration of CNS and ENS is commonly seen as a part of aging and aging-related disease. Recent studies have shown evidence linking sirtuins and various metabolic pathways like aging, obesity, and diabetes through the modulation of various hormones like leptin, ghrelin, melatonin, and serotonin, thereby influencing neurodegeneration in ENS and CNS.

Through our review paper, we have tried to emphasize the concept of microbiota–gut–brain axis, a special focus on dysbiosis, and its role in the development of neurodegenerative disorders (AD and PD), IBD, IBS, and the therapeutic importance of drugs targeting sirtuins thereby modulating the above-mentioned axis. Sirtuins may contribute towards the regulation of gut microbiota, and interventions to correct dysbiosis may help in slowing down the progression of disorders. 

Prebiotics are non-digestible dietary fibers that promote the growth and activity of beneficial bacteria in the gut. These substances serve as a food source for specific beneficial gut bacteria. When these bacteria ferment prebiotics, they produce short-chain fatty acids (SCFAs) and other metabolites. Probiotics are live beneficial bacteria or yeasts that can provide health benefits when consumed in adequate amounts. By directly introducing beneficial microbes into the gut, probiotics can help restore the gut microbiome’s balance. They can also produce bioactive compounds and interact with the gut lining, immune system, and nervous system, potentially influencing brain function and behavior. Prebiotics pass through the small intestine without being fully digested and reach the large intestine, where they serve as a food source for beneficial gut bacteria.

For example, beneficial gut bacteria ferment the prebiotics and produce short-chain fatty acids (SCFAs) and other metabolites. These SCFAs can positively influence the gut lining, reduce inflammation, and impact the gut’s immune response. Additionally, some of these metabolites may be able to enter the bloodstream. Meanwhile, the same individual takes a probiotic supplement containing live strains of beneficial bacteria like Lactobacillus and Bifidobacterium. These probiotics are able to survive the harsh conditions of the digestive tract and populate the gut, restoring a healthy balance of gut microbes.

The presence of these beneficial gut bacteria from the probiotics can also influence the gut–brain axis. They may produce neurotransmitters such as serotonin and GABA, which can impact mood and behavior. Moreover, gut bacteria can interact with the gut’s immune system and indirectly affect the brain’s immune responses. The combined effect of prebiotics and probiotics on the gut microbiome-brain axis may lead to improvements in mood, reduced stress levels, and enhanced cognitive function in this individual [145].

## 4. Conclusions

The microbiota–gut–brain axis has been closely studied over the past few decades, leading to more understanding of its effects. This review aimed to present the ENS and CNS interlinkage, describing the gut microbiome playing a key role in the growth and maintenance of ENS and CNS and how it is referred to as the second brain influencing gut–microbiota–brain axis under both physiological and pathological circumstances. Moving forward, it will provide more insight into the field of preventive gastroenterology. Then, it centralized the attention to CNS disorders with ENS implications, with special emphasis on neurodegenerative diseases like Parkinson’s disease, Alzheimer’s disease, multiple sclerosis, and other neurological disorders. Nutrition and cognition are also discussed with details of neurologic involvement in IBD, IBS, GI functional disorders, as well as stress-induced GI disorders. Future studies should aim to dissect and identify gut–brain access at the molecular level with much more detail. Clinical studies and trials are needed to precisely dissect the molecular pathways. Of key significance would be what specific microbiome metabolites reach the CNS, what they exactly do at that level, and through which mechanism. Establishing this association will open up new possibilities for various therapeutic strategies to combat the most debilitating disorders faced by society today.

## Figures and Tables

**Table 1:** Inflammatory markers are associated with various neurological conditions.

Si No	Disease	Findings
1	Multiple Sclerosis	a. Caspase-1 and ASC (apoptosis-associated speck-like protein) in serum are predictors for the severity of the disease [124]. b. IL-1β, Caspase-1 ↑, IL-18, in CSF and PBMC (Peripheral Blood Mononuclear Cells) [125].
2	Alzheimer’s Disease	a. IL-18, Caspase-1, ASC, and IL-1β, in PBMCs. b. IL-1β, IL-18 in astrocytes, microglia, and neurons surrounding Aβ plaques [126,127].
3	Parkinson’s disease	a. Caspase-1, IL-1β in the striatum and serum [128].
4	Neuropsychiatric disorder	a. Increased activity of NLRP3 inflammasome [129,130,131].

↑—Increased serum levels.

## Data Availability

The review was based on publicly available academic literature databases.
